# Liver X Receptor: A Novel Therapeutic Target

**DOI:** 10.4103/0250-474X.41445

**Published:** 2008

**Authors:** M. B. Patel, N. A. Oza, I. S. Anand, S. S. Deshpande, C. N. Patel

**Affiliations:** Department of Pharmacology, Shri Sarvajanik Pharmacy College, Near Arvind Baug, Mehsana - 384 001, India

**Keywords:** Liver X Receptors, Retinoid X Receptors, PPARs, GLUT1, SMRT

## Abstract

The liver X receptors α and β are orphan nuclear receptors that are key regulators in maintaining cholesterol homeostasis. Originally they were found to play an important role in reverse cholesterol transport, a pathway for the removal of excess cellular cholesterol. However several groups have now shown that the liver X receptors also functions in lipid and carbohydrate metabolism, cellular differentiation, apoptosis and many immune responses. Tissue distribution of the two paralogues differs with liver X receptor β ubiquitously expressed, while liver X receptor α is confined to the liver, kidney, intestine, spleen, adipose tissue, macrophages and skeletal muscle. The endogenous ligands for the liver X receptors are certain oxidized derivatives of cholesterol, the oxysterols. Upon activation by oxysterols, the receptors form obligate heterodimers with retinoid X receptors α, β and γ; and become competent to activate the transcription of target genes.

Liver X receptor-α (LXRα) and LXRβ (also known as NR1H3 and NR1H2, respectively) were cloned more than a decade ago based on sequence homology with other receptors. LXRs were originally considered orphan nuclear receptors, because their natural ligands were unknown^1^,^2^; however, these receptors were “adopted” following the discovery that metabolites of cholesterol, oxysterols bind to and activate these receptors at physiological concentrations^2^,^3^. LXRα is highly expressed in the liver and at lower levels in the adrenal glands, intestine, adipose, macrophages, lung, and kidney, whereas LXRβ is ubiquitously expressed^4^. The LXRs are ligand-dependent transcription factors that form permissive heterodimers with the retinoid X receptor (RXR); i.e., the complex can be activated by ligands of either partner. LXR/RXR heterodimers bind to LXR-responsive elements (LXREs) in DNA consisting of direct repeats (DRs) of the core sequence AGGTCA separated by 4 nucleotides (DR-4)^5^. Like most other nuclear receptors that form heterodimers with RXR, LXRs reside within the nucleus, bound to cognate LXREs and in complex with corepressors such as silencing mediator of retinoic acid and thyroid hormone receptor (SMRT)^6^ and nuclear receptor corepressor (N-CoR)^7^. In the absence of ligand, these corepressor interactions are maintained and the transcriptional activity of target genes is repressed. Binding of ligand to LXR results in a conformational change that facilitates coactivator-for-corepressor-complex exchange and transcription of target genes^8^,^9^. Ligand activation of LXRs also inhibits transcription from promoters of certain genes (e.g., proinflammatory cytokines) that do not contain LXREs, a phenomenon referred to as *trans*-repression. This review will focus on recent advances in our understanding of the roles of LXRs in physiology and homeostasis as well as the links between LXR action and metabolic diseases such as atherosclerosis.

## Liver X Receptors (LXRs):

The LXR subfamily consists of two members namely, LXRα (NR1H3) and LXRβ (NR1H2)^10^,^11^. LXRα is highly expressed in liver tissue, with lower levels present in adipose tissue, intestine, kidney and spleen whereas LXRβ is ubiquitously expressed^12^. Although cholesterol loading results in activation of genes with LXR response elements, neither free cholesterol nor cholesteryl esters (CEs) appear to be physiological ligands for LXRs. Recent studies have shown that oxysterols are specific ligands for the LXRs. The most potent oxysterols include: 24(S),25-epoxycholesterol, which is produced in hepatocytes and macrophages, 24(S)-hydroxycholesterol, an abundant cholesterol metabolite in brain tissue and 22(R)-hydroxycholesterol, an intermediate in steroid hormone production^12^. The 27-hydroxycholesterol, which is produced in macrophages in response to cholesterol loading, also activates LXRs and has been proposed to be an important physiological ligand^13^.

## Autoregulation of LXRα:

Many nuclear receptors autoregulate their own expression. Recently, an autoregulatory function has been described to the human LXRα gene^14^. This regulation is mediated through an LXRE in the distal portion of the LXRα gene promoter that confers responsiveness to both LXRα and LXRβ. Although this region is conserved in both the mouse and human promoter, autoregulation is only observed in human cells, suggesting that humans may be more responsive to LXRα agonists than mice. Interestingly, one study identified LXRα autoregulation exclusively in macrophages^15^, whereas a second study has found autoregulation in adipocytes and hepatocytes.

## Regulation of LXRα by PPARs:

In addition to autoregulatory control, recent studies have shown that the expression of LXRα is also regulated by members of another family of nuclear receptors, the peroxisome proliferator-activated receptors (PPARs). Two subtypes of the PPARs, PPARα and PPARγ are particularly interesting in that they have been well characterized and are involved in lipid metabolism. PPARα is abundantly expressed in liver, kidney, heart, and muscle, and regulates genes involved in fatty acid catabolism and lipoprotein production. PPARγ is selectively expressed in adipose tissues and governs adipogenesis. Both PPAR subtypes are found in macrophages that are associated with atherosclerotic plaques^16^. PPARs promote cholesterol efflux from macrophages through an ABCA1 pathway, possibly by mediating expression of LXRα^16^. A PPAR response element (PPRE) has been identified in both the mouse and human LXRα gene. Although one study claims that both PPARα and PPARγ agonists can induce LXRα expression in macrophages^17^, a second study shows that only PPARγ can upregulate expression of LXRα through the PPRE^17^. It has also been reported that PPARα agonists induce the expression of LXRα in rat liver.

## Regulation of LXRβ^17^:

Interestingly, no regulation of LXRβ has been observedso far in any studies, implicating distinct roles of LXRα and LXRβ in certain regulatory pathways. The major LXRs are enlisted in Table 1^18^–^36^.

**TABLE 1:** LXR TARGET GENES

Gene	Function of gene product	Gene expression response to LXR activation in	
		
		Humans	Mice
ABCA1	Cellular cholesterol efflux and HDL formation	Induced^18^–^20^	Induced^18^,^21^
SREBP-1c	Master regulator of fatty acid synthesis	Induced^22^	Induced^23^,^24^
CYP7A1	Rate determining enzyme of bile acid synthesis	No effect^25^–^27^	Induced^24^,^28^
CETP	Transfer of neutral lipids between lipoprotein particles	Induced^29^	Gene not present^30^
PLTP	Phospholipid transfer; lipoprotein assembly	Weakly induced	Induced^31^
LXRα	Sensor of cholesterol excess	Induced^32^,^33^	No effect^22^,^34^
ABCG1	Unknown	Strongly induced^35^	Induced^35^
ABCG5/ABCG8	Efflux of plant sterols and cholesterol into bile and intestinal lumen	Not known	Induced^36^

Table shows various target genes for LXR in humans as well as in mice and their final gene products

## LXR and cholesterol metabolism:

The initial identification of oxysterols as physiological ligands of LXRs pointed to a possible role for these receptors in cholesterol metabolism^3^,^37^. Conclusive evidence for this notion came from studies of LXRα deficient (*Lxra^−/−^*) mice, which display marked cholesteryl ester accumulation in their livers when challenged with a cholesterol-rich diet^38^. This phenotype led to the identification of Cyp7a1, a member of the cytochrome p450 family of enzymes and the rate-limiting enzyme in the classical pathway of bile acid synthesis, as the first direct target of LXRs. The inability of *Lxra^−/−^* mice to induce hepatic *Cyp7a1* expression results in a diminished ability to metabolize cholesterol to bile acids, and the accumulation of cholesteryl esters. Interestingly, the LXRE found in the promoter of rodent *Cyp7a1* is not conserved in humans^39^. LXRβ is also expressed in the liver, but *Lxrb^−/−^* mice do not display an obvious hepatic phenotype even when challenged with a high-cholesterol diet^40^, indicating that LXRα is likely to be the dominant isoform in this tissue.

Subsequent studies demonstrated that LXRs also regulate a set of genes that participate in the process of reverse cholesterol transport the transport of excess cholesterol in the form of HDL from peripheral tissue to the liver^41^. *In vivo* activation of LXRs with a synthetic, high-affinity ligand increases HDL levels and net cholesterol secretion^42^. To a large extent these activities are dependent on the ability of LXRs to control the expression of members of the ABC superfamily of membrane transporters^43^, including ABCA1^42^,^44^, ABCG5, ABCG8^45^,^46^ and ABCG1^47^–^49^. Mutations in the *ABCA1* gene are the cause of Tangier disease, a rare disorder that is characterized by the virtual absence of HDL in plasma of afflicted patients, the accumulation of cholesterol in tissue macrophages, and an increased incidence of cardiovascular disease^50^–^52^. It is now well established that ABCA1 facilitates the efflux of cholesterol and phospholipids to lipid-poor lipoproteins (e.g., apoA-I), and its induction may contribute to the increase in plasma HDL levels seen with LXR ligand treatment. The ability of LXR ligands to decrease intestinal absorption of cholesterol^42^ appears to be mediated by induction of two other ABC transporters, ABCG5 and ABCG8^45^,^46^. These apically localized transporters form a functional heterodimer that acts to limit cholesterol and plant-sterol absorption in the gut, and to mediate cholesterol efflux from hepatocytes into bile. Mutations in either of the heterodimer partners cause the rare genetic disease sitosterolemia, which is characterized by increased absorption of plant sterols and premature atherosclerosis.

## LXR and intermediary metabolism:

**Fig. 1:**
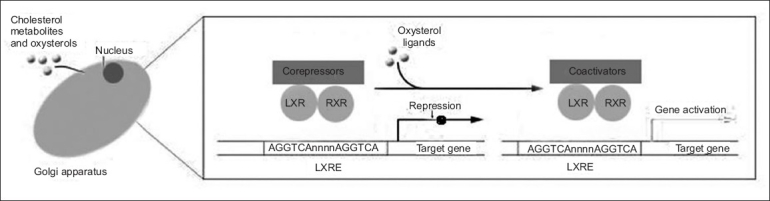
LXRs and cholesterol-sensing transcription factors. Within the nucleus, LXR/RXR heterodimers are bound to LXREs in the promoters of target genes and in complex with corepressors (e.g., smrt, n-cor). In response to the binding of oxysterol ligands, the corepressor complexes are exchanged for coactivator complexes, and target gene expression is induced (adopted from Zelcer and Tontonoz^75^).

In addition to their ability to modulate cholesterol metabolism, LXRs are also key regulators of hepatic lipogenesis. Treatment of mice with synthetic LXR agonists elevates triglyceride levels in the liver as well as transiently in the plasma, an effect that poses a significant obstacle to the development of these compounds as human therapeutics^53^,^54^. The lipogenic activity of LXRs results from the upregulation of the master regulator of hepatic lipogenesis SREBP-c^55^, as well as induction of fatty acid synthase^54^, acyl-CoA carboxylase, and stearoyl-CoA desaturase I. LXRs also positively regulate several enzymes involved in lipoprotein remodeling, including lipoprotein lipase, human cholesteryl ester transport protein (CETP), and the phospholipid transfer protein (PLTP). It is likely that some of the effects of activated LXR on plasma lipoproteins are mediated through action of these enzymes. The role of cholesterol metabolites in the regulation of LXRs is depicted in fig. 1.

Within the nucleus, LXR/RXR heterodimers are bound to LXREs in the promoters of target genes and in complex with corepressors (e.g., smrt, n-cor). In response to the binding of oxysterol ligands, the corepressor complexes are exchanged for coactivator complexes, and target gene expression is induced. As outlined above, LXRs are intrinsically involved in key metabolic pathways, but the integrated action of LXRs on whole-body energy balance has been only recently addressed. Kalaany *et al*.^56^,^57^ found that LXR-null mice are resistant to obesity when challenged with a diet high in both fat and cholesterol. Remarkably, this phenotype was dependent on the presence of cholesterol in the diet and was largely attributed to increased peripheral utilization of dietary fat as manifested by a marked enhanced metabolic rate. Moreover, an increase in expression and activity of deiodinase 2 (Dio2), an enzyme that generates active thyroid hormone (T3) from its inactive form (T4), was seen in livers of LXR-null mice fed the cholesterol-containing diet and would be predicted to increase hepatic utilization of dietary fat as well. The mechanism by which dietary cholesterol signals change in systemic metabolism is not yet known. Regardless, this study emphasizes the central role of LXRs as regulators of fat storage and utilization^58^.

## LXR and macrophage cholesterol metabolism:

In addition to their essential role in innate immunity, macrophages are central to the development of the atherosclerotic lesion because of their ability to take up modified lipoproteins and to release inflammatory mediator^59^,^60^. Within the lesion, macrophages are postulated to accumulate ligands of LXRs by several distinct pathways. Uptake of modified lipoproteins may provide the cell with preformed oxysterol activators of LXR. Ligands may also be generated intracellularly from accumulated cholesterol by action of the mitochondrial *Cyp27*^61^. Although *Cyp27* is not a direct target of LXRs, its enzymatic product 27-hydroxycholesterol is an LXR ligand^62^,^63^. Additionally, the intracellular production of 24-(*S*), 25-epoxycholesterol, a potent naturally occurring LXR ligand, in rodent and human macrophages was reported recently^64^. Increasing the levels of this metabolite by partially inhibiting the enzyme, 2,3-oxidosqualene lanosterol cyclase results in increased LXR transcriptional activity. A primary function of LXRs in macrophages is to maintain cellular cholesterol homeostasis. Activation of LXRs in lipid-loaded macrophages leads to induction of genes involved in the cholesterol efflux pathway in an attempt to reduce the intracellular cholesterol burden. The ABC transporters discussed above are critical for the ability of LXRs to enhance efflux to cholesterol acceptors. Expression of ABCA1 is strongly induced by natural and synthetic LXR ligands as well as by loading of cells with modified lipoproteins. This induction has been attributed to the presence of LXREs in the proximal promoter of the *ABCA1* gene^42^,^44^. LXRs are in fact essential for lipid-inducible ABCA1 expression, as induction is lost in macrophages from *Lxrab* double-knockout mice (*Lxrab^−/−^* mice). Conversely, LXRs are unable to stimulate cholesterol efflux to lipid-poor lipoproteins in fibroblasts from Tangier disease patients, demonstrating that ABCA1 is essential for the LXR-mediated efflux pathway. The importance of ABCA1 for atherogenesis is underscored by the fact that macrophage-specific loss of this gene results in increased lesion formation in murine models^65^,^66^.

ABCG1, another member of the ABC transporter family, is also strongly induced by cholesterol loading of macrophages^49^ and was recently identified as a direct target of LXRs in mouse and human cells^47^,^48^. Induction of ABCG1 may provide an additional pathway for cholesterol efflux from macrophages or may act in concert with ABCA1. ABCG1 is thought to function as a homodimer^67^. Using *in vitro* assays ABCG1 has been demonstrated to facilitate cholesterol efflux to HDL-2 and 3 particles, but not to apoA-I, thus distinguishing it mechanistically from ABCA1^67^,^68^. At present, however, the cellular localization of ABCG1 is not defined, and it is therefore unclear whether ABCG1 directly mediates efflux to HDL particles or facilitates this process by influencing intracellular cholesterol trafficking. In line with the latter possibility is a recent study demonstrating that activation of LXRs in human macrophages boosts cholesterol trafficking to the plasma membrane at the expense of esterification^69^.

**Fig. 2:**
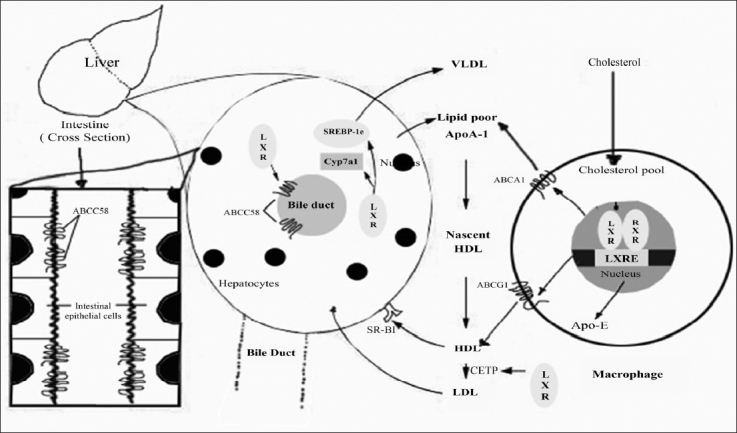
Role of LXRs in reverse cholesterol transport from macrophages. The uptake of modified lipoproteins by macrophages results in increased LXR transcriptional activity and efflux of cholesterol to lipid poor apoA-I by ABCA 1 and to HDL by ABCG1. In humans but not in mice, introduction of CETP expression transfers lipid from HDL to LDL. Once HDL/LDL is taken up by the liver, LXR promotes net cholesterol excretion (adopted from Zelcer and Tontonoz^75^).

The generation and initial characterization of *Abcg1^−/−^* mice has revealed striking phenotypes that point to a critical function for this transporter in whole-body lipid homeostasis^68^. In support of *in vitro* experiments, macrophages lacking ABCG1 showed a diminished cholesterol efflux capacity to HDL. Cholesterol efflux to apoA-I, which is mainly mediated by ABCA1, was unchanged, however. In accordance with these findings, lipid-laden macrophages were detected in the lungs and liver of *Abcg1*-null mice after 9 weeks of a high-fat and -cholesterol diet. Remarkably, this phenotype was not accompanied by changes in the profile of plasma lipoproteins. It is tempting to speculate that ABCG1 activity, like ABCA1 activity, would be antiatherogenic, but this remains to be tested directly. The closely related protein ABCG4 is also modestly induced in macrophages by cholesterol loading and by LXR ligands and has been reported to promote cholesterol efflux to HDL particles when overexpressed in HEK293 cells^67^,^70^. Studies of the physiological roles of this transporter are eagerly awaited. An additional mechanism that may contribute to the LXR-driven reverse cholesterol transport is the induction of a subset of apolipoproteins that may serve as cholesterol acceptors. Specifically, LXRs induce *Apoe* gene expression in macrophages and adipose tissue, but not in the liver^71^. Additionally, the *Apoc* gene cluster (*ApocI*, *ApocII*, and *ApocIV*) is also induced by LXRs in macrophages, and *Apod* is a target for LXR in adipose tissue. The significance of the induction of the *Apoc* cluster and of *Apod* by LXR for lipoprotein metabolism is at present unknown. In contrast, the protective role of *Apoe* in atherogenesis is well established. Loss of macrophage apoE leads to increased lesions, whereas overexpression of apoE in these cells is protective. More recently, LXR was shown to directly regulate hepatic, but not intestinal, apoA-4 in mouse liver, and apoA-4 in the human HepG2 cell line. In humans, plasma levels of apoA-4 are inversely correlated with cardiovascular disease. Whether apoA-4 is regulated in macrophages by LXR is at present unknown. apoA-5 is the only apolipoprotein known to be repressed by LXRs^72^. Repression of hepatic apoA-5 by LXR is not direct but appears to be secondary to induction of SREBP-1c. As increased levels of apoA-5 are strongly correlated with reduced plasma triglycerides^73^, repression by LXR may contribute to the hypertriglyceridemic effects of synthetic LXR agonists^31^. The role of LXRs in reverse cholesterol transport from macrophages is shown in (fig. 2).

The uptake of modified lipoproteins by macrophages results in increased LXR transcriptional activity and efflux of cholesterol to lipid-poor apoA-I by ABCA1 and to HDL by ABCG1. In humans, but not mice, induction of CETP expression transfers lipid from HDL to LDL. Once HDL/LDL is taken up by the liver, LXR promotes net cholesterol excretion. In rodents, but not humans, LXR induces expression of Cyp7a1, which initiates the conversion of cholesterol into bile acids. LXRs also induce cholesterol secretion into bile through the transporters ABCG5 and ABCG8. In the intestine, apical ABCG5 and ABCG8 also act to limit dietary cholesterol uptake.

## LXR and lipid homeostasis:

Lipid absorption from the intestinal lumen into the villi, the metabolism of lipoproteins in the plasma and the movement of lipids out of macrophages or the liver. Genes that are activated by LXR are shown in green.

## LXR and macrophage inflammatory signaling:

Activation of inflammatory signaling pathways and release of inflammatory mediators are fundamental to the diverse immune functions of macrophages. The microenvironment present within the atherosclerotic lesion is proinflammatory and results in activation of these same pathways. A substantial number of studies demonstrate that excessive inflammation within the arterial wall is a risk factor for cardiovascular disease and promotes atherogenesis^59^,^60^. Therefore, factors that act to limit inflammation in this setting may prove to be beneficial in reducing disease progression. Considerable evidence has emerged to indicate that, in addition to inducing genes involved in reverse cholesterol transport, LXRs reciprocally repress a set of inflammatory genes after bacterial, LPS, TNF-α, or IL-1β stimulation. Examples of such genes include those involved in generation of bioactive molecules such as iNOS and COX2, IL-6 and IL-1β, the chemokines monocyte chemoattractant protein-1 (MCP-1) and MCP-3. LXR ligands repress these genes in macrophages derived from *Lxra^−/−^*, and *Lxrb^−/−^* mice but are unable to do so in macrophages from *Lxrab^−/−^* mice, indicating that both LXR isoforms possess antiinflammatory activity. Subsequent work has suggested that tissue factor and *osteopontin*, both inflammatory genes associated with an increased risk for developing atherosclerosis, are subject to similar repression by LXR ligands in macrophages. Inhibition of inflammatory signaling by LXR is not limited to isolated macrophages but also manifests itself *in vivo*. Experiments in several different models have confirmed the antiinflammatory effects of LXRs. When challenged intraperitoneally with LPS, *Lxrab^−/−^* mice exhibit an exacerbated systemic inflammatory response and increased hepatic expression of iNOS, TNF-α, and IL-1β. Synthetic LXR agonists also reduce inflammation in a model of irritant contact dermatitis. A similar result was reported found that LXR ligands showed activity comparable to that of a steroid-based drug in an oxazolone-induced allergic dermatitis model. Furthermore, administration of LXR ligands to mice inhibits tissue factor expression in the kidney and lung after an LPS challenge. Finally, in 2 mouse models of chronic atherogenic inflammation, *Apoe^−/−^* and *Ldlr^−/−^* mice, administration of LXR ligands repressed the aortic expression of MMP9 and tissue factor while inducing expression of ABCA1.

**Fig. 3:**
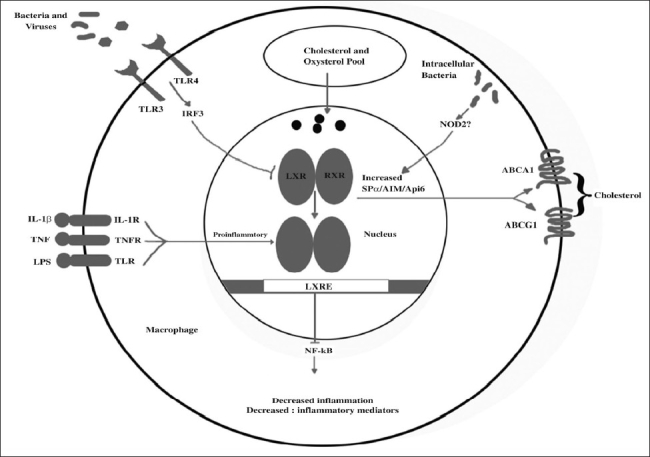
Integration of lipid metabolic and inflammatory signaling in macrophages by LXRs. Recognition of cytokines, bacterial components, or intact pathogens by their corresponding receptors initiates expression of proinflammatory genes (e.g., iNOS). Activation of the TLR3/4 receptors by these signals blocks LXR-dependent gene transcription and cholesterol efflux from macrophages via an IFN regulatory factor 3-dependent (IRF3-dependent) pathway. On the other hand, ligand activation of LXRs inhibits NF-κB-dependent induction of inflammatory gene expression. Intracellular bacteria induce LXRα expression, possibly through a NOD2-dependent pathway, and promote macrophage survival, through induction of Api6 (also known as AIM and SPα) and other targets (adopted from Zelcer and Tontonoz^75^).

The mechanism underlying the repression of inflammatory genes by LXRs is poorly understood. LXREs have not been identified in the proximal promoters of the repressed genes; this points to an indirect mechanism. In addition to possible competition for transcriptional coactivators, the body of evidence suggests that inhibition of the NF-κB pathway is involved. Inhibition of this pathway does not entail inhibition of NF-κB translocation to the nucleus, binding to DNA, or degradation of the NF-κB inhibitor IκB. Most likely, *trans*-repression of NF-κB by LXR involves a nuclear event. In a recent study of *trans*-repression of the *iNOS* promoter by PPARγ, sumoylation of PPARγ was identified as a possible mechanism involved in this process. Participation in both metabolic and inflammatory control is a common feature of a number of different nuclear receptor signaling pathways. For example, Ogawa *et al.*^76^ demonstrated recently that LXR, PPARγ, and the glucocorticoid receptor repress an overlapping yet distinct set of inflammatory genes in a stimulus-dependent manner. In addition to pointing out the possibility of treating inflammatory-related diseases in a combinatorial approach that targets 2 or more of these receptors, this study also underscores the complexity of inflammatory gene regulation and the likelihood that these nuclear receptors have unique functions within the immune system. (fig. 3) shows integration of lipid metabolic and inflammatory signaling in macrophages by LXRs.

Recognition of cytokines, bacterial components, or intact pathogens by their corresponding receptors initiates expression of proinflammatory genes (e.g., iNOS). Activation of the TLR3/4 receptors by these signals blocks LXR-dependent gene transcription and cholesterol efflux from macrophages via an IFN regulatory factor 3-dependent (IRF3-dependent) pathway. On the other hand, ligand activation of LXRs inhibits NF-κB-dependent induction of inflammatory gene expression. Intracellular bacteria induce LXRα expression, possibly through a NOD2-dependent pathway, and promote macrophage survival, through induction of Api6 (also known as AIM and SPα) and other targets.

## LXR and carbohydrate metabolism^74^^,75^:

LXR also plays an important role in regulation of carbohydrate metabolism. In liver, treatment of LXR agonist significantly inhibits expression of gluconeogenetic enzymes such as (the rate-limiting enzyme in gluconeogenesis), fructose biphosphatase 1, and glucose-6-phosphatase resulting in a dramatic reduction of plasma glucose observed by treating diabetic rodents with an LXR agonist. It also increases insulin sensitivity in insulin-resistant rats and that gluconeogenic genes were suppressed, leading to decreased hepatic glucose output. LXRα increases basal uptake of glucose in adipocytes, mediated mainly through the GLUT1 transporter. An LXR agonist was shown to induce expression of GLUT1 mRNA as well as protein levels. LXR have also reported to cause increased expression of GLUT4 mRNA.

## Metabolic effects of LXR:

**Fig. 4:**
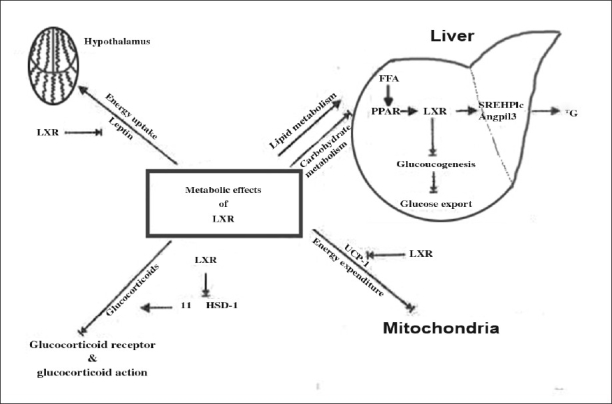
Metabolic effects of LXRs. Figure shows that LXRS are involved in suppressing glucocorticoid effects, which might lead to reduced development of obesity and improved insulin sensitivity linked with glucocorticoid activity. Furthermore, LXR could decrease gluconeogenesis, as previously described, and indirectly by inhibiting glucocorticoid stimulatory effects on PEPCK (gluconeogenic enzyme) expression (adopted from Zelcer and Tontonoz^75^).

UCP-l is a target gene of LXR. Leptin and UCP-1 expression was downregulated after administration of an LXR agonist. So decreased leptin levels would eventually lead to increased energy intake and decreased UCP-l leads to decreased energy expenditure leading to obesity. Both a synthetic and a natural LXR agonist decreased 11β-HSDl mRNA expression and activity by 50%. This suggests an indirect effect of LXR on 11β-HSDl expression, probably by stimulating expression of an inhibitor or by inhibiting expression of an activator of 11β-HSDl. Moreover, long-term treatment of mice with a synthetic LXR agonist down regulated 11β-HSDl mRNA expression in both brown adipose tissue and liver of wild-type mice but not in LXRα ^−/−^β^−/−^ mice. The inhibition of 11β-HSDl indicates that LXR might be involved in suppressing glucocorticoid effects, which might lead to reduced development of obesity and improved insulin sensitivity linked with glucocorticoid activity. Furthermore, LXR could decrease gluconeogenesis, as previously described, and indirectly by inhibiting glucocorticoid stimulatory effects on PEPCK (gluconeogenic enzyme) expression. Taken together, these findings indicate a possible role of LXR in the hormonal regulation of overall energy metabolism. Major metabolic effects induced by LXRs are shown in (fig. 4).

## CONCLUSIONS

LXR- member of Nuclear Receptor Family is activated by certain oxysterol derivatives of cholesterol. They play an important role in cholesterol, lipid, and carbohydrate metabolism. LXRα is highly expressed in liver tissue. They respond to elevated cholesterol levels via transactivation of genes involved in sterol transport (ABCA1, ABCG1, ABCG5, and ABCG8), cholesterol efflux and high-density lipoprotein (HDL) metabolism, and sterol catabolism (CYP7A1). They also play a central role in regulating cellular lipid content through activation of SREBP-1c, which is the master regulator of de novo lipogenesis. LXRs were found to upregulate angiopoietin- like protein 3 (Angpf13), a member of the family of vascular endothelial growth factors that is also a key regulator of lipid metabolism. LXRs induce reverse cholesterol transport from peripheral tissue to liver via HDL by stimulating the production of apolipoproteins and ABC transporters. LXRs, however, also induce fatty acid production and serum triglycerides by stimulating expression of SREBPlc and Angpt13, leading to an increased risk of developing metabolic diseases such as type 2 diabetes. In atherosclerosis-prone mouse models, LXR agonist treatment leads to increased HDL levels and decreased formation of atherogenic lesions. A dramatic reduction of plasma glucose has been observed by treating diabetic rodents with an LXR agonist. LXR agonist increases insulin sensitivity in insulin-resistant rats and that gluconeogenic genes were suppressed, leading to decreased hepatic glucose output. LXR agonist was shown to induce expression of GLUT1 mRNA as well as GLUT4 mRNA. Leptin and UCP-1 expression was downregulated after administration of an LXR agonist. Both a synthetic and a natural LXR agonist decreased 11β-HSDl mRNA expression and activity by 50%. The inhibition of 11β-HSDl indicates that LXR might be involved in suppressing glucocorticoid effects, which might lead to reduce development of obesity and improved insulin sensitivity. CETP and lipoprotein lipase (LPL) genes are directly activated by oxysterols and LXR. So, LXR play important role in Lipoprotein Metabolism. Oxysterols activate the liver orphan receptors (LXR) to induce cholesterol 7α-hydroxylase and ATP-binding cassette family of transporters and thus promote reverse cholesterol transport from the peripheral tissues to the liver for degradation to bile acids. The livers X receptors are critical for the control of lipid homeostasis. LXRs serve as cholesterol sensors that regulate the expression of multiple genes involved in the efflux, transport, and excretion of cholesterol. Synthetic LXR agonists inhibit the development of atherosclerosis in murine models. These observations identify the LXR pathway as a potential target for therapeutic intervention in human cardiovascular disease.
